# Pregnancy and Perinatal Outcomes of Patients With Prior Cesarean Section After a Single Embryo Transfer in IVF/ICSI: A Retrospective Cohort Study

**DOI:** 10.3389/fendo.2022.851213

**Published:** 2022-05-23

**Authors:** Lin Wang, Jing Wang, Nan Lu, Jiayin Liu, Feiyang Diao

**Affiliations:** State Key Laboratory of Reproductive Medicine, Clinical Center of Reproductive Medicine, The First Affiliated Hospital of Nanjing Medical University, Nanjing, China

**Keywords:** live birth, single embryo transfer, *in vitro* fertilization, Cesarean delivery, Cesarean section defect

## Abstract

**Objective:**

To study the influence of the previous cesarean section on the pregnancy outcomes and perinatal outcomes in single embryo transfer (SET) cycles in an *in vitro* fertilization/intracytoplasmic sperm injection-embryo transfer (IVF/ICSI-ET) setting compared to those with previous vaginal delivery (VD). In addition, the association between fertility outcomes and different cesarean scar defect (CSD) sizes was studied.

**Method:**

This was a retrospective cohort study conducted in the Reproductive Center of the First Affiliated Hospital of Nanjing Medical University. A total of 4,879 patients with previous delivery history undergoing SET were included between January 2015 and April 2019. Patients were divided into the VD group and cesarean delivery (CD) group according to different modes of previous delivery. The primary outcome was live birth rate. The pregnancy outcomes of CD were analyzed as a subgroup and the relationship between pregnancy outcomes as well as the different sizes of CSD were explored by logistic regression analysis.

**Results:**

There were no significant differences in live birth rate, clinical pregnancy rate, and miscarriage rate between the CD group and VD group. The incidence rates of pregnancy complications such as pregnancy hypertension, gestational diabetes mellitus, placenta abnormalities, premature rupture of membrane, and postpartum hemorrhage were similar in the two groups. Live birth rate was significantly lower in the CSD group (23.77% vs 37.01%, aOR: 0.609, 95% CI: 0.476-0.778) comparing to patients without CSD. There were also significant differences in clinical pregnancy rate (37.52% vs 47.64%, aOR: 0.779, 95%CI: 0.623-0.973) and miscarriage rate (34.55% vs 20.59%, aOR: 1.407, 95%CI:1.03-1.923). Large size CSD significantly decreased live birth rate (13.33% vs 26.29%, aOR: 0.422, 95%CI: 0.197-0.902) and clinical pregnancy rate (25.33% vs 40.09%, aOR: 0.503, 95%CI: 0.272-0.930) compared with small size CSD.

**Conclusion:**

For women with previous cesarean sections, the pregnancy outcomes were similar to those with previous VD without increased perinatal complications following SET. The presence of CSD was associated with a marked reduction in live birth rate, especially in patients with large size CSD.

## 1 Introduction

In recent decades, the prevalence of cesarean section (CS) in the global scope grew two-fold, increasing from 12% in 2000 to 21% in 2015 in all deliveries (1). In China, the percentage of CS delivery increased from 28.8% to 36.7% from 2008 to 2018 (2), which was much higher than the reasonable range of 10-15% recommended by the World Health Organization (WHO). There are rising concerns regarding the short-term complications and long-term risks of CS, including placental implantation and uterine rupture in the next pregnancy (3). It is well established that pregnancy risks dramatically increase with twin pregnancies than singleton pregnancies (4), especially in patients with scarred uterus (5). There is an urgent need to reduce the multiple pregnancies rate in patients with the previous cesarean delivery(CD) (6). However, the incidence of multiple pregnancies increases in patients undergoing IVF by multiple embryos transfer to achieve a higher pregnancy rate (7). Single embryo transfer (SET) is an effective strategy to avoid multiple pregnancies without compromising the cumulative live birth rates compared with double embryos transfer (8). Previous studies showed SET not only decreased multiple pregnancies risk but also improved the perinatal outcomes compared with singletons resulting from double-embryo transfers (9). Hence, SET is recommended for patients with a scarred uterus. Keeping that in mind, the pregnancy and perinatal outcomes after SET in patients with previous CS are still unknown.

A significantly lower rate of natural conception after CD was reported (10). Several studies investigated the association of prior CD and pregnancy outcomes in IVF cycles. The conclusions have been controversial as these studies lack homogeneity and different studies evaluated different numbers (one or more) of embryos transfer including a mix of cleavage-stage and blastocyst-stage embryos transfer. It is important to further explore the effect of previous CD on pregnancy outcomes in a SET setting.

Cesarean scar defect (CSD) is also called niche or diverticulum, which refers to poor healing of uterine scar after CS (11). Its prevalence varies from 6.9-69% depending on the study population and methodology used (12). Some reports suggested that CSD impaired embryo implantation and subsequent fertility (13, 14). Residual myometrial thickness (RMT) measured less than 3mm is defined as large CSD (15) with a high risk of spontaneous uterine rupture (16, 17). No published studies have investigated the relationship between pregnancy outcomes with different sizes of CSD.

Therefore, this study aimed to investigate the impact of previous CD compared with previous vaginal delivery (VD) on the reproductive outcomes and perinatal outcomes in patients undergoing SET. We also explored the relationship between the pregnancy outcomes and different CSD sizes in patients undergoing IVF treatment.

## 2 Material and Method

### 2.1 Study Population

This retrospective study was conducted at the Department of Assisted Reproduction Center of the First Affiliated Hospital of Nanjing Medical University from January 2015 to April 2019. Patients included into this study had at least one previous delivery (including CS and VD) and SET was performed. Only the first embryo transfer was included in the analysis. According to the previous modes of delivery, patients were divided into two groups: the previous CD group and the previous VD group. Exclusion criteria was: advanced maternal age (>43years); recurrent pregnant loss: two or more pregnancy loss before 24 weeks of gestation; untreated mild to severe hydrosalpinx, endometriosis, uterine adhesion; Preimplantation Genetic Testing (PGT) cycles; and oocytes donation cycles.

### 2.2 CSD Evaluation

All patients were assessed by Voluson E8 ultrasound system (General Electric Voluson, 2014, USA) equipped with a 5-9 MHz three-dimensional transvaginal probe. The three-dimensional-transvaginal ultrasound (3D-TVS) was taken 3-7 days after menstruation. CSD is defined as a wedge-shaped anechoic area with an indentation of the myometrium larger than 2 mm at the site of CS. The depth, width of CSD, and RMT were measured in the sagittal plane (18). The large CSD was estimated as RMT less than 3mm, middle size CSD was RMT in a range of 3-6 mm, small size CSD was defined as RMT more than 6mm.

### 2.3 Treatment Protocol

#### 2.3.1 Ovarian Stimulation

Conventional gonadotropin releasing hormone (GnRH) agonist (GnRHa) (midluteal GnRHa suppression) and GnRH antagonist (antagonist administration when the leading follicle diameter reaches 13mm) regimens were performed for ovarian stimulation. The initial dose of recombinant follicle-stimulating hormone (FSH) was 100–300 IU/day depending on age, body mass index (BMI), ovarian reserve, and possible response to stimulation.

#### 2.3.2 Ovulation Trigger and Luteal Phase Support

When at least the diameter of two follicles reached 18 mm or three follicles greater than 17mm, a single bonus of 6500 IU recombinant human chorionic gonadotropin (hCG) injection was administered subcutaneously and oocyte retrieval was performed 36 hours later. Only one embryo was transferred 3–5 days after oocyte retrieval. The luteal phase was daily supported by progesterone from the day of oocyte retrieval and continued for 14 days after the embryo transfer. In the cases of potential severe ovarian hyperstimulation syndrome, all embryos were frozen.

#### 2.3.3 Frozen-Thawed Embryo Transfer (FET) Protocol

Endometrial preparation for FET was performed by four regimens, including natural cycle, induced ovulation cycle, hormone replacement therapy (HRT), and GnRHa combined HRT (GnRHa+HRT) regimen. The natural cycle was performed in women with regular menstruation with or without hCG trigger. An induced ovulation cycle was conducted among anovulatory women with letrozole in combination with human menopausal gonadotropin (hMG). Luteal phase support was administered on the day of ovulation. For the HRT cycle, exogenous estrogen was administered until the endometrium reached optimal thickness, then the supplement of exogenous progesterone was performed. The GnRHa+HRT was mainly for women with endometriosis or adenomyosis. Pituitary down-regulation was achieved by a full dose of GnRHa 3.75mg at day 1 or day 2 of the menstrual cycle and HRT was performed 25-28 days later. A cleavage-stage or a blastocyst-stage embryo was transferred 3-5 days after endometrial development with progesterone.

### 2.4 Outcome Measures

The primary outcome was live birth rate, defined as live births after 28 gestational weeks. The secondary outcome parameters included biochemical pregnancy, clinical pregnancy, miscarriage, ectopic pregnancy, twin pregnancies, neonatal outcomes, and maternal pregnancy complications. Biochemical pregnancy was detected as positive serum hCG 14 days after embryo transfer. Clinical pregnancy was defined as the presence of a gestational sac with or without fetal heart detected by the ultrasound examination at the eighth gestational week. Miscarriage referred to pregnancy loss before 28 gestational weeks. Ectopic pregnancy referred to the gestational sac detected out of the uterine cavity. Twin pregnancies was defined as two fetal heartbeats detected by ultrasound. The interested maternal complications included gestational hypertension, gestational diabetes, placental abnormalities such as placenta previa and placental abruption, premature rupture of the membrane, and postpartum hemorrhage. Neonatal outcomes comprised preterm birth (<37 weeks of gestation), stillbirth (fetal death after 28 gestational weeks), low birth weight (< 2500g), and very low birth weight (<1500g).

### 2.5 Statistical Analysis

Data were analyzed by SPSS statistics (version 26; IBM, Armonk, NY). Continuous variables were described as mean values with standard deviation and categorical variables were described as numbers with percentages. Propensity score matching (PSM) was applied to balance the distributions of observed baseline characteristics between the CD groups and the VD groups with a 1:1 nearest-neighbor matching strategy and caliper was set as 0.2. Age, BMI, infertility diagnosis, fertilization methods, fresh or frozen-thawed cycle, the protocol of fresh and frozen embryo transfer, and endometrial thickness on the day of embryo transfer were selected as the matching factors. After PSM, Student’s t-test or Mann-Whitney U test was used for continuous variables, depending on the normality of the data distribution. Fisher’s exact test and Pearson’s χ2 were used for categorical data. Univariate and multivariate logistic regression analysis was performed to test the relationship between the presence of CSD and reproductive outcomes. The adjusted covariates of logistic regression included age, BMI, fresh or frozen-thawed cycle, the stage of embryo transferred, and endometrial thickness on the day of transfer. The association of the different sizes of CSD and the reproductive outcomes were performed by the logistic regression by the adjusted factors described above. The crude and adjusted results were expressed as odds ratio (OR) with 95% confidence intervals (CIs). A two-sided P value of less than 0.05 was considered statistically significant.

## 3 Results

### 3.1 General Information of Patients With Different Delivery Modes Following SET

**Figure 1:**
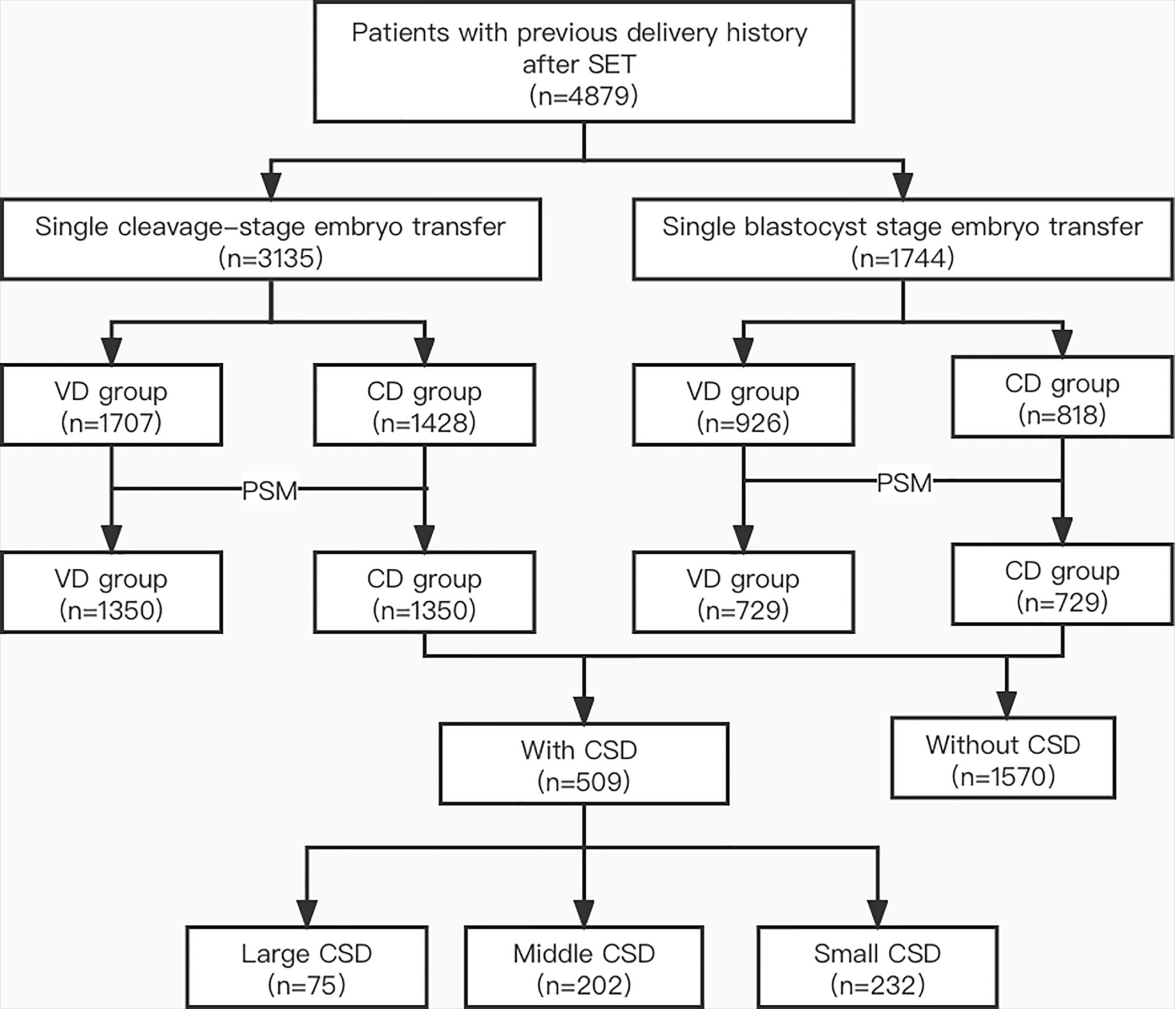
Flow chart of the study. SET, single embryo transfer; VD, vaginal delivery; CD, cesarean delivery; PSM, propensity score matching; CSD, cesarean scar defect.

As shown in **Figure 1**, 3,135 women who underwent single cleavage-stage embryo transfer and 1,744 women who underwent single blastocyst-stage embryo transfer were included. Before matching, the baseline characteristics such as age, BMI, and the thickness of the endometrium were not balanced in VD and CD groups. After subsequent propensity score matching, 1,350 patients were assigned to the VD and CD groups, respectively, in patients with single cleavage-stage embryo transfer and 729 patients were included in each group with single blastocyst-stage embryo transfer. The baseline variables such as age, BMI, infertility factors, endometrial thickness, the protocol of fresh and frozen embryo transfer were all comparable between the VD and CD groups in both cleavage-stage and blastocyst-stage embryo transfer populations (all *P*>0.05) (**Table 1**).

**Table 1:** Demographics and cycle characteristics of patients with different previous delivery modes.

	Cleavage-stage embryo						Blastocyst -stage embryo					
	before PSM			after PSM			before PSM			after PSM		
	VD (n=1707)	CD (n=1428)	*P* value	VD (n=1350)	CD (n=1350)	*P* value	VD (n=926)	CD (n=818)	*P* value	VD (n=729)	CD (n=729)	*P* value
Age (year)	35.52±4.71	36.81±5.00	<0.001^*^	36.52±4.96	36.3±4.64	0.237	33.48±4.76	33.39±4.25	0.707	33.43±4.77	33.30±4.41	0.58
BMI (kg/m2)	23.13±2.78	23.63±2.79	0.004^*^	23.13±2.82	23.25±2.96	0.283	22.38±2.73	23.68±2.81	0.028^*^	22.38±2.74	22.63±2.7	0.079
Infertility diagnosis, n (%)												
Tubal factors	597 (34.97)	469 (32.84)	0.294	438 (32.44)	446 (33.04)	0.613	460 (49.68)	402 (49.14)	0.976	376 (51.58)	363 (49.19)	0.898
Decreased ovarian reservation	551 (32.28)	448 (31.37)		402 (29.78)	426 (31.56)		195 (21.06)	169 (20.66)		149 (20.44)	154 (20.87)	
Unexplained infertility	157 (9.20)	135 (9.45)		125 (9.26)	118 (8.74)		95 (10.26)	85 (10.39)		61 (8.37)	60 (8.13)	
Combined factors	402 (23.55)	376 (26.33)		385 (28.52)	360 (26.67)		176 (19.01)	162 (19.8)		143 (19.62)	152 (20.6)	
Fertilization method, n (%)												
IVF	1371 (80.32)	1139 (79.76)	0.699	1090 (80.74)	1065 (78.89)	0.231	745 (80.45)	687 (83.99)	0.055	599 (82.17)	602 (82.58)	0.837
ICSI	336 (19.68)	289 (20.24)		260 (19.26)	285 (21.11)		181 (19.55)	131 (16.01)		130 (17.83)	127 (17.42)	
transfer cycle, n (%)												
Fresh	720 (42.18)	602 (42.16)	0.990	526 (38.96)	575 (42.59)	0.055	40 (4.32)	45 (5.50)	0.253	24 (3.29)	28 (3.84)	0.572
Frozen	987 (57.82)	826 (57.84)		824 (61.04)	775 (57.41)		886 (95.68)	773 (94.50)		705 (96.71)	701 (96.16)	
Stimulation protocol, n (%)												
Agonist	371 (51.53)	319 (52.99)	0.596	277 (52.66)	305 (53.04)	0.899	32 (80)	35 (77.78)	0.802	22 (91.67)	20 (71.43)	0.065
Antagonist	349 (48.47)	283 (47.01)		249 (47.34)	270 (46.96)		8 (20)	10 (22.22)		2 (8.33)	8 (28.57)	
Endometrial preparation method, n (%)												
Natural cycle	435 (44.07)	375 (45.40)	0.314	384 (46.6)	352 (45.42)	0.272	357(40.29)	305 (39.46)	0.292	270 (38.30)	280 (39.94)	0.641
Induced ovulation	302 (30.60)	272 (32.93)		246 (29.85)	261 (33.69)		350 (39.50)	299 (38.68)		275 (39.01)	277 (39.51)	
Hormone replacement treatment (HRT)	169 (17.12)	119 (14.41)		135 (16.38)	106 (13.68)		108 (12.19)	87 (11.25)		93 (13.19)	77 (10.98)	
GnRHa+HRT	81 (8.21)	60 (7.26)		59 (7.16)	56 (7.23)		71 (8.01)	82 (10.61)		67 (9.50)	67 (9.56)	
Endometrial thickness (mm)	9.62±1.89	9.50±1.70	0.040^*^	9.56±1.85	9.49±1.79	0.296	9.76±1.74	9.59±1.64	0.040^*^	9.70±1.68	9.65±1.67	0.505

PSM, propensity score matching; VD, vaginal delivery; CD, cesarean delivery; BMI, body mass index; IVF, in vitro fertilization; ICSI, intracytoplasmic sperm injection; GnRHa, gonadotropin-releasing hormone agonist; Values are described as mean ± standard deviation or number (percentage); ^*^P<0.05.

### 3.2 The Pregnancy and Perinatal Outcomes of VD and CD Groups

The pregnancy outcomes of the VD and CD groups are presented in **Table 2**. The biochemical pregnancy rate (cleavage-stage: 40.67% vs 39.18%, *P*=0.432; blastocyst-stage: 71.60% vs 69.41%, *P*=0.358), clinical pregnancy rate (cleavage-stage: 36.22% vs 34.29%, *P*=0.295; blastocyst-stage: 66.67% vs 65.29%, *P*=0.543), and live birth rate (cleavage-stage: 26.59% vs 23.70%%, *P*=0.084; blastocyst-stage: 57.20% vs 52.40%, *P*=0.066) were higher in the VD groups but the differences failed to reach significant difference. In addition, no significant differences were observed in miscarriage rate, ectopic pregnancy rate, or twin pregnancies rate in different groups. **Table 3** shows the perinatal outcomes including maternal complications and neonatal outcomes. The prevalence rate of preterm birth, low birth, very low birth, and obstetric complications did not differ in VD and CD groups (all *P*>0.05). However, we observed a statistically significant decrease in gestational weeks of delivery in patients with previous CS (cleavage-stage: 38.39 ± 1.89 weeks vs 38.08 ± 1.55 weeks, *P*=0.02; blastocyst-stage: 38.36 ± 1.63 weeks vs 37.95 ±1.49 weeks, *P*<0.001).

**Table 2:** Pregnancy outcomes of patients with different previous delivery modes.

Parameter	Cleavage-stage embryo			Blastocyst-stage embryo		
	VD group (n=1350)	CD group (n=1350)	*P* value	VD (n=729)	CD (n=729)	*P* value
Biochemical pregnancy rate, %(n/N)	40.67 (549/1350)	39.18 (529/1350)	0.432	71.60 (522/729)	69.41 (506/729)	0.358
Clinical pregnancy rate,%(n/N)	36.22 (489/1350)	34.29 (463/1350)	0.295	66.67 (487/729)	65.29 (476/729)	0.543
Miscarriage rate, %(n/N)	24.13 (118/489)	28.94 (134/463)	0.093	13.78 (67/487)	18.07 (86/476)	0.067
Ectopic pregnancy rate, %(n/N)	2.86 (14/489)	1.94 (9/463)	0.356	0.62 (3/487)	1.68 (8/476)	0.120
Twin pregnancies rate, %(n/N)	1.02 (5/489)	0.86 (4/463)	0.179	1.64 (8/487)	0.63 (3/476)	0.139
Live birth rate, %(n/N)	26.59 (359/1350)	23.70 (320/1350)	0.084	57.20 (417/729)	52.40 (382/729)	0.066

VD, vaginal delivery; CD, cesarean delivery. Values are described as percentage (number/total number),^*^P<0.05.

**Table 3:** Perinatal outcomes of patients with different modes of previous delivery.

Parameter	Cleavage-stage embryo			Blastocyst -stage embryo		
	VD group (n=359)	CD group (n=320)	*P* value	VD (n=417)	CD (n=382)	*P* value
Maternal complications, n (%)	43 (11.98)	47 (14.69)	0.299	41 (9.83)	52 (13.61)	0.096
Gestational diabetes mellitus, n (%)	18 (5.01)	24 (7.50)	0.179	26 (6.24)	28 (7.33)	0.538
Gestational hypertension, n (%)	11 (3.06)	8 (2.50)	0.656	3 (0.72)	5 (1.31)	0.403
Placenta previa, n (%)	8 (2.23)	8 (2.50)	0.816	4 (0.96)	9 (2.36)	0.119
Placental abruption, n (%)	1 (0.28)	0 (0.00)	0.345	1 (0.24)	0 (0.00)	0.338
Premature rupture of membrane, n (%)	4 (1.11)	6 (1.88)	0.411	6 (1.44)	7 (1.83)	0.660
Postpartum hemorrhage, n (%)	1 (0.28)	1 (0.31)	0.935	1 (0.24)	3 (0.79)	0.275
Neonatal outcomes						
Gestational age of delivery (weeks)	38.39±1.89	38.08±1.55	0.020^*^	38.36±1.63	37.95±1.49	<0.001^*^
Birth weight (g)	3497.78±614.20	3418.38±539.95	0.646	3481.08±513.40	3408.96±536.82	
Preterm birth, n (%)	35 (9.75)	34 (10.63)	0.379	34 (8.15)	41 (10.73)	0.212
Stillbirth, n (%)	2 (0.55)	0 (0.00)	0.182	0 (0.00)	1 (0.26)	0.317
Low birth weight (<2500g), n (%)	10 (5.29)	13 (4.06)	0.400	9 (2.16)	13 (3.40)	0.283
Very low birth weight (<1500g), n (%)	1 (0.28)	2 (0.62)	0.462	1 (0.24)	1 (0.26)	0.950

VD, vaginal delivery; CD, cesarean delivery. Values are described as mean±standard deviation or number (percentage) ^*^P<0.05.

### 3.3 The Baseline Characteristics and Logistic Regression Analysis of Reproductive Outcomes Between Previous CD Patients With and Without CSD

As shown in **Table 4**, the number of patients with previous CD without visible scars was 1,570 and the number of patients with CSD was 509. The baseline characteristics such as age, BMI, endometrial thickness, and the proportion of blastocyst-stage transfer were comparable between the patients with and without CSD (all *P*>0.05), while the proportion of fresh embryo transfer was significantly different between the two groups (34.77% vs 27.13%, *P*=0.001). We investigated the pregnancy outcomes by logistic regression to overcome the imbalance and the results are shown in **Table 5**. After adjusting for age, BMI, fresh or frozen-thawed cycle, the stage of embryo transferred, endometrial thickness, the live birth rate was significantly lower in patients with CSD than those without CSD (23.77% vs 37.01%, aOR: 0.609, 95%CI: 0.476-0.778). The probability of clinical pregnancy rate (37.52% vs 47.64%, aOR: 0.779, 95%CI: 0.623-0.973) also decreased in patients with CSD. A significantly increased risk of miscarriage was observed in the CSD group (34.55% vs 20.59%, aOR: 1.407, 95%CI: 1.03-1.923). There were no significant differences in biochemical pregnancy rate or ectopic pregnancy rate.

**Table 4:** Baseline characteristics of patients with and without CSD.

Item	Without CSD group (n=1570)	CSD group (n=509)	*P* value
Ages (years)	35.04±4.71	35.84±4.99	0.339
BMI (kg/m2)	22.85±2.86	23.56 ±2.87	0.240
Endometrial thickness(mm)	9.59 ±1.73	9.49 ±1.79	0.522
Blastocyst-stage embryo transfer rate, %(n/N)	36.18% (568/1570)	31.63% (161/509)	0.062
Fresh embryo transfer rate, %(n/N)	27.13% (426/1570)	34.77% (177/509)	0.001^*^

CSD, cesarean section defect; BMI, body mass index; Values are described as mean±standard deviation or percentage (number/total number); ^*^P<0.05.

**Table 5:** Logistic regression analysis of reproductive outcomes of patients with and without CSD.

Parameter	Without CSD group (n=1570)	CSD group (n=509)	Crude OR	*P* value	Adjusted OR	*P* value
Biochemical pregnancy rate, % (n/N)	51.78 (813/1570)	43.61 (222/509)	0.720 (0.589-0.881)	0.001^*^	0.865 (0.696-1.076)	0.194
Clinical pregnancy rate, % (n/N)	47.64 (748/1570)	37.52 (191/509)	0.660 (0.538-0.810)	0.001^*^	0.779 (0.623-0.973)	0.027^*^
Miscarriage rate, % (n/N)	20.59 (154/748)	34.55 (66/191)	1.370 (1.007-1.863)	0.045^*^	1.407 (1.030-1.923)	0.032^*^
Ectopic pregnancy rate, % (n/N)	1.74 (13/748)	2.09 (4/191)	0.949 (0.308-2.923)	0.927	1.088 (0.349-3.389)	0.885
Live birth rate, % (n/N)	37.01 (581/1570)	23.77 (121/509)	0.531 (0.422-0.667)	<0.001^*^	0.609 (0.476-0.778)	<0.001^*^

CSD, cesarean section defect. Values are described as percentage (number/total number); Adjusted for age, BMI, fresh or frozen-thawed cycle, the stage of embryo at transfer, endometrial thickness; ^*^P<0.05.

### 3.4 The Relationship Between the Reproductive Outcomes and Different CSD Size

After adjusting for important confounders (age, BMI, fresh or frozen-thawed cycle, the stage of embryo transferred, endometrial thickness), patients with large CSD were associated with a significantly lower live birth rate (13.33% vs 26.29%, aOR: 0.422, 95%CI: 0.197-0.902) compared with patients with small CSD. Similarly, biochemical pregnancy rate (32.00% vs 45.69%,aOR: 0.546, 95%CI: 0.305-0.978) and clinical pregnancy rate(25.33% vs 40.09%, aOR: 0.503, 95%CI: 0.272-0.93) were significantly lower in the large CSD group. However, there were no significant differences observed in miscarriage rate among patients with different sizes of CSD (**Table 6**). 

**Table 6:** Logistic regression analysis of patients with different sizes of CSD.

Parameter	(%) (n/N)	Crude OR (95%CI)	*P* value	Adjusted OR (95%CI)	*P* value
**Biochemical pregnancy rate**					
Small CSD	45.69 (106/232)	reference		reference	
Middle CSD	45.54 (92/202)	0.994 (0.681-1.452)	0.976	0.884 (0.586-1.334)	0.557
Large CSD	32.00 (24/75)	0.559 (0.323-0.969)	0.038^*^	0.546 (0.305-0.978)	0.042^*^
**Clinical pregnancy rate**					
Small CSD	40.09 (93/232)	reference		reference	
Middle CSD	39.11 (79/202)	0.960 (0.653-1.412)	0.836	0.862 (0.567-1.310)	0.488
Large CSD	25.33 (19/75)	0.507 (0.283-0.908)	0.022^*^	0.503 (0.272-0.930)^*^	0.028^*^
**miscarriage rate**					
Small CSD	31.18 (29/93)	reference		reference	
Middle CSD	35.44 (28/79)	1.126 (0.645-1.967)	0.675	1.105 (0.620-1.967)	0.735
Large CSD	47.37 (9/19)	0.955 (0.430-2.120)	0.909	1.012 (0.450-2.278)	0.976
**Live birth rate**					
Small CSD	26.29 (61/232)	reference		reference	
Middle CSD	24.75 (50/202)	0.922 (0.598-1.422)	0.714	0.832 (0.522-1.326)	0.439
Large CSD	13.33 (10/75)	0.431 (0.208-0.892)	0.023^*^	0.422 (0.197-0.902)	0.026^*^

CSD, cesarean section defect; Values are described as percentage ( number/total number ); Adjusted for age, BMI, fresh or frozen-thawed cycle, stage of embryo transferred, endometrial thickness;^*^P<0.05.

## 4 Discussion

CS rate is increasing worldwide and continues to grow. CS leads to an anatomic change of the uterus and contributed to a lower rate of childbearing (9, 19). Recent studies attempt to demonstrate the relation between CD and subsequent pregnancy outcomes in IVF, but the results have been controversial. The underlying cause of the difference was considered to be the heterogeneity of these studies. One of the factors was the imbalanced baseline characteristics of the patients, including maternal age, endometrial thickness at the day of transfer, and BMI. Zhang et al. (20) observed no difference in live birth rate (40.59% vs 45.38%, *P*=0.466) between the CD and VD groups, however, there was imbalanced maternal age. Diao (21) et al. also revealed no significant difference in live birth rate (33.1% vs36.4%, OR: 0.86, 95%CI: 0.64~1.16, *P*>0.05) with thinner endometrial thickness in the CD group. In another study, Friedenthal J et al. (22) reported nearly a 10% reduction in the live birth rate of the CD group with imbalanced BMI. Our preliminary data also showed some imbalanced characteristics including higher age, larger BMI, and thinner endometrium in the CD group. To overcome this imbalance, we utilized PSM and reported a lower live birth rate, clinical pregnancy rate, and higher miscarriage rate without a statistically significant difference in women with previous CD compared with women with previous VD following SET. Furthermore, previous studies included patients with a mix of cleavage-stage and blastocyst-stage embryo transfers in different proportions, which could lead to a biased interpretation of the results. In a prospective study performed by Patounakis et al. (23), the live birth rate (39% vs 32%, *P*=0.366) was similar between different modes of the previous delivery with 35-39% blastocyst-stage embyro transfer rate. When the blastocyst transfer rate was only 7.9-9% [Huang et al. (24)], an obviously lower live birth rate (27.5% vs 33.4%, *P*=0.03) in patients with previous CD was discovered. Previous work demonstrates that blastocyst-stage embryo transfer was associated with an increased pregnancy rate than cleavage-stage embryo transfer (25, 26), so we further stratified patients with different stages of embryo development, respectively, to avoid bias. The results showed the same trend of lower live birth rate regardless of cleavage-stage embryo or blastocyst-stage embryo transfer.

Patients with previous CD history had an increased risk of life-threatening pregnancy complications with the subsequent twin gestation than singleton pregnancy (27). SET was defined as a multiple birth minimization strategy (28). Our data shows comparable perinatal outcomes following SET between patients with different previous delivery modes. The incidences of adverse obstetric and neonatal outcomes did not show significant differences between the CD and VD groups. The twin pregnancies rates were 0.63-1.64% in patients with CD history following SET. In contrast to our study, some studies (29, 30) transferred one or more embryos in patients with previous CS, and the twin birth rate approximately reached 30% with significantly higher preterm birth rate than singleton births. Some patients even received selective fetal reduction to decrease the risk of adverse events in twin birth. Selective fetal reduction was an invasive procedure complicated with infection and miscarriage (31) and SET was more likely to be the first option to achieve a healthy live birth. Moreover, the CD group showed a significantly lower gestational age than VD group (cleavage-stage: 38.39 ± 1.89 weeks vs 38.08 ± 1.55 weeks, *P*=0.02, blastocyst-stage: 38.36 ± 1.63 weeks vs 37.95 ± 1.49 weeks, *P*<0.001). This might be associated with the timing of elective repeat CS without labor. Most repeat cesarean deliveries were performed around 37-39 weeks of gestation (32) in patients with previous history of CS concerning maternal and neonatal safety (33).

The presence of CSD had a negative effect on subsequent fertility (34). In this study, the presence of CSD shows a detrimental effect on subsequent pregnancy. The results remained robust after adjusting for the possible confounders and effect-modifying factors. Patients with CSD were associated with a significantly lower rate of subsequent live birth (aOR: 0.609, 95%CI: 0.476~0.778, *P*<0.001) and clinical pregnancy (aOR: 0.779, 95%CI: 0.623~0.973, *P*=0.027), as well as a higher likelihood of miscarriage (aOR:1.407, 95%CI: 1.03~1.923, *P*=0.032) compared with those without defect at the site of the cesarean incision. The results were in agreement with previous studies (21, 35). The existence of CSD could lead to poor pregnancy outcomes in patients undergoing IVF.

In literature, large CSD (RMT<3mm) in non-pregnant women is regarded as a high risk of uterine dehiscence or rupture in subsequent pregnancies (36). However, there is no definitive classification of CSD to predict the pregnancy outcomes in IVF. This study explored the relationship between different sizes of scar defects and pregnancy outcomes with a logistic regression model adjusted for potential confounding factors. Live birth rate (13.33% vs 26.29%, aOR: 0.422, 95%CI: 0.197-0.902) and clinical pregnancy rate (25.33% vs 40.09%, aOR: 0.503, 95%CI: 0.272-0.930) sharply decreased in patients with large CSD compared with those with small CSD. The underlying mechanisms appear to be associated with reduced scar contractility around the fibrotic scar (37). The impaired ability of myometrium cannot expel the blood completely in the niche with degradation of hemoglobin (38). The fluid accumulated at the CS site may hamper the embryo implantation like in patients with hydrosalpinx (39). The toxic environment with excess iron might disturb the endometrial receptivity and uterine microbiota (40). Another explanation is that CSD may compromise the process of decidualization (41). The delayed endometrial maturation has a negative effect on steroid receptor expression and impairs embryo implantation (42). Furthermore, the altered immune microenvironment in the scar can lead to a decline in fertility with less vascularization and leukocytes (13).

The major weakness of our study was its retrospective design. We were unable to get more detailed previous information about the CS, such as previous pregnancy complications, emergent or elective CS, single or double-layer suture of the uterus and the ability to assess the role of related information on pregnancy outcomes was not available. Another limitation was the sensitivity of 3D-TVS examination for CSD. Saline contrast sonography, hysteroscopy, or magnetic resonance imaging (MRI) might provide more accuracy but were also more invasive and expensive (43, 44). A better diagnosis tool and classification for CSD needs to be explored.

## 5 Conclusion

This study demonstrated no significant differences in pregnancy outcomes and no higher incidences of perinatal complications in patients with different modes of previous delivery in SET cycles. Further subgroup analyses suggested the presence of CSD was associated with a lower live birth rate, and large CSD was identified as the main deleterious factor for live birth. Our findings suggest clinicians should assess the healing of uterus scars and inform patients of the adverse impacts of CSD in the subsequent pregnancy.

## Data Availability Statement

The raw data supporting the conclusions of this article will be made available by the authors, without undue reservation.

## Ethics Statement 

The studies involving human participants were reviewed and approved by Reproductive Medical Ethics Committee of the first affiliated hospital of Nanjing Medical University. The patients/participants provided their written informed consent to participate in this study.

## Author Contributions

LW and FD conceived and designed the study. JW collected data and performed the statistical analysis. LW wrote the first draft which was revised by NL and JW. The study was supervised by JL and FD. All the authors contributed to the study and approved the submitted version.

## Funding

This work was supported by National Key Research and Development Program of China (2018YFC100258, 2017YFC1001303) and National Natural and Science Foundation of China (81730041).

## Conflict of Interest

The authors declare that the research was conducted in the absence of any commercial or financial relationships that could be construed as a potential conflict of interest.

## Publisher’s Note

All claims expressed in this article are solely those of the authors and do not necessarily represent those of their affiliated organizations, or those of the publisher, the editors and the reviewers. Any product that may be evaluated in this article, or claim that may be made by its manufacturer, is not guaranteed or endorsed by the publisher.
